# Homeodomain-Interacting Protein Kinase-2: A Critical Regulator of the DNA Damage Response and the Epigenome

**DOI:** 10.3390/ijms17101638

**Published:** 2016-09-27

**Authors:** Yuki Kuwano, Kensei Nishida, Yoko Akaike, Ken Kurokawa, Tatsuya Nishikawa, Kiyoshi Masuda, Kazuhito Rokutan

**Affiliations:** Department of Pathophysiology, Institute of Biomedical Sciences, Tokushima University Graduate School, 3-18-15 Kuramoto-cho, Tokushima 770-8503, Japan; knishida@tokushima-u.ac.jp (K.N.); c201411001@tokushima-u.ac.jp (Y.A.); ken.kurokawa178@gmail.com (K.K.); nishikawa.tatsuya@tokushima-u.ac.jp (T.N.); kiyoshim@tokushima-u.ac.jp (K.M.); rokutan@tokushima-u.ac.jp (K.R.)

**Keywords:** DNA damage responses, HIPK2, HP1γ, cell cycle, apoptosis

## Abstract

Homeodomain-interacting protein kinase 2 (HIPK2) is a serine/threonine kinase that phosphorylates and activates the apoptotic program through interaction with diverse downstream targets including tumor suppressor p53. HIPK2 is activated by genotoxic stimuli and modulates cell fate following DNA damage. The DNA damage response (DDR) is triggered by DNA lesions or chromatin alterations. The DDR regulates DNA repair, cell cycle checkpoint activation, and apoptosis to restore genome integrity and cellular homeostasis. Maintenance of the DDR is essential to prevent development of diseases caused by genomic instability, including cancer, defects of development, and neurodegenerative disorders. Recent studies reveal a novel HIPK2-mediated pathway for DDR through interaction with chromatin remodeling factor homeodomain protein 1γ. In this review, we will highlight the molecular mechanisms of HIPK2 and show its functions as a crucial DDR regulator.

## 1. Introduction

Cells are constantly subjected to DNA damage events caused by environmental or endogenous genotoxic agents [1,2]. Metabolic by-products, such as reactive oxygen species (ROS), are probably the most frequent intrinsic source of DNA damage. Extrinsic or environmental sources of DNA damage include ultraviolet (UV) light, ionizing radiation (IR), and exposure to numerous chemotherapeutic drugs, such as cisplatin, Adriamycin, and roscovitine [3,4]. Many studies have shown that DNA-damaging agents promote genome instability, which is a main driving force of tumorigenesis [5,6,7]. Eukaryotic cells rely on a strictly coordinated series of events, termed the DNA damage response (DDR), to cope with genotoxic insults to maintain homeostasis. The DDR includes cell-cycle checkpoint activation, regulation of DNA replication, and DNA damage repair [8,9]. Recent studies have shown that the DDR functions as a potent intracellular regulator of transformation and tumorigenesis [5,10,11]. In fact, dysregulation of DDR affects the progression of clinically pertinent diseases, including premature aging, immunological disorders, neurodegenerative diseases, and cancer.

Although molecular mechanisms of DDR are still not fully understood, numerous DDR factors have been identified as tumor suppressors that prevent cancer progression [5,12]. Homeodomain-interacting protein kinase 2 (HIPK2) is a DNA damage-responsive serine/threonine kinase that activates the apoptotic program through interaction with diverse downstream targets, including tumor suppressor p53 [13,14]. In DDR, HIPK2 modulates the activity of several proteins thorough site-specific phosphorylation in response to the DNA damage. To date, epigenetic factors related to chromatin remodeling and histone modification regulate the integrated response of chromatin for promoting DNA damage-triggered signaling and DNA repair [15]. Here, we summarize the molecular mechanisms of the HIPK2-mediated DDR and suggest a possible role of this kinase as an epigenetic regulator linking DNA repair.

## 2. Signaling Pathways for the DNA Damage Response (DDR)

**Figure 1:**
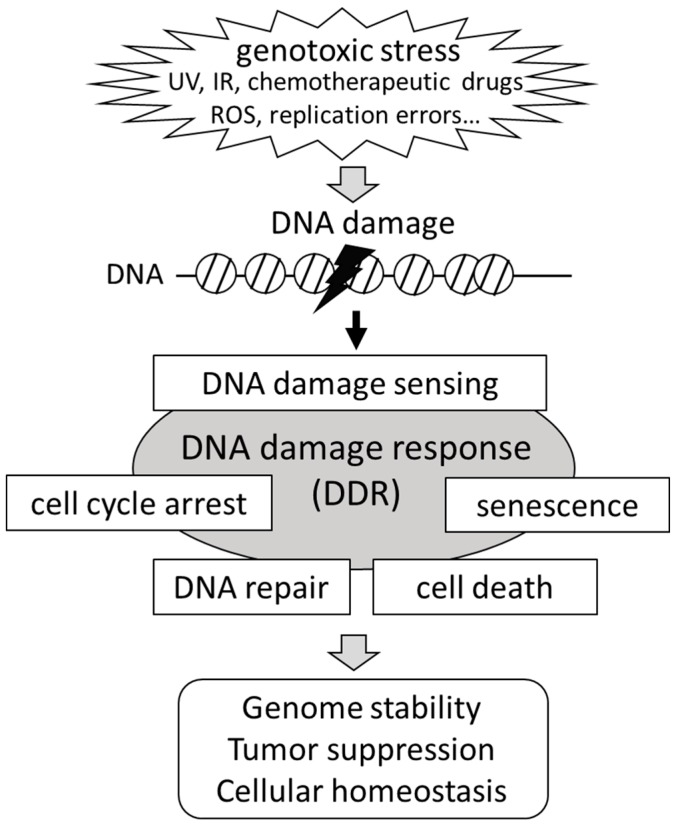
Schematic representation of the DNA damage response (DDR). Cells are constantly exposed to various environmental or endogenous genotoxic agents. Through the DDR pathway, the DNA damage is recognized by sensor protein complexes. The accumulation of DNA damage provokes various cellular events, including cell cycle arrest, DNA repair, cellular senescence, or cell death. The DDR plays a key role in the genomic stability that protects cells from tumorgenesis. UV, ultraviolet; IR, ionizing radiation; ROS, reactive oxygen species.

Once cells are exposed to environmental or cell-derived stressors, like UV light or ROS, these insults can cause DNA lesions, such as single-strand breaks (SSBs) or double-strand DNA breaks (DSBs) [5,16]. The DDR employs appropriate cellular machinery to detect DNA damage and maintain genomic integrity. The DDR in damaged cells provokes various cellular events, including cell cycle arrest, DNA repair, cellular senescence, or cell death. While cellular senescence and cell cycle arrest of the damaged cells block proliferation, cell death signaling facilitates removal of the cells that are irreversibly damaged [10] (Figure 1). In general, DDR pathways consist of multi-regulated steps: initial detection of DNA damage, recruitment of DNA repair factors to the damaged lesions and DNA repair.

DSBs constitute the most deleterious form of DNA lesions. DSBs are caused spontaneously during DNA replication when arrested replication forks collapse [17,18]. In addition, extrinsic or environmental sources of DNA damage, such as ultraviolet-C (UV-C), IR, and genotoxic drugs, can trigger DSBs [1,2,3,4]. In mammalian cells, DSBs are primarily repaired by two different pathways, homologous recombination (HR) and the non-homologous end-joining (NHEJ) pathway [5]. HR repair is a relatively accurate and efficient pathway, but it depends on the remains of the undamaged sister chromatid DNA, while the NHEJ pathway is not affected by the presence of replicated DNA [19,20,21,22]. The DSB repair pathway is tightly controlled during the cell cycle through DNA damage-induced signaling. If the damage is unrepaired or aberrantly repaired, DSBs latently lead to chromosomal rearrangements and aberrations, resulting in developmental and neurodegenerative disorders, and cancer [2,5]. NHEJ is the crucial mechanism for DNA repair of IR-induced DNA damage. A homeodomain-containing protein, HOXB7, interacts with DNA-dependent protein kinase holoenzymes, including Ku70, Ku80, the catalytic subunit of DNA-PK (DNA-PK_cs_), and poly (adenosine diphosphate (ADP)) ribose polymerase, which, in turn, enhances NHEJ by stimulating DNA-PK activity and contributes to DNA damage repair in response to IR [23].

DNA damage is sensed by specified protein complexes that are directly bound to the site of the DNA lesion. Then, the sensor complexes activate master DDR Ser/Thr kinases. These kinases belong to the family of phosphatidylinositide 3-kinase (PI3K)-like kinases and they constitute the apex of the DNA damage signaling cascade. DSBs are sensed by the heterotrimeric Mre11/Rad50/NBS1 (MRN) complex [24]. The MRN complex is recruited to DSBs and triggers auto-phosphorylation of the DNA damage checkpoint kinase ataxia telangiectasia mutated (ATM) at Ser1981 to switch from inactive dimers to active monomers [25,26]. The Rad9-Hus1-Rad1 (9-1-1) clamp interacts with ATM and Rad3-related (ATR) and promotes its activation in response to collapsed DNA replication forks and replication stress [27,28]. The activation of ATM or ATR subsequently mediates the phosphorylation of downstream substrates that are implicated in DNA repair, cell cycle arrest, and cell death.

## 3. Homeodomain-Interacting Protein Kinase 2 (HIPK2)

**Figure 2:**
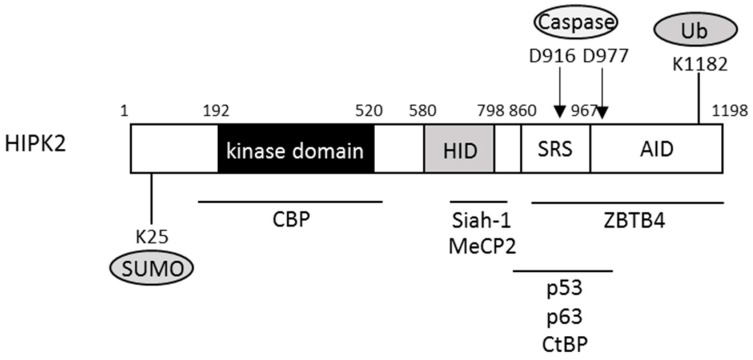
Schematic summary of homeodomain-interacting protein kinase 2 (HIPK2) structure. The N-terminal domain contains the sumoylation sites (K25). D916 and D977 are caspase-6 cleavage sites. K1182 is the ubiquitylation site. Some HIPK2-interacting proteins are shown. SUMO, sumoylation; Ub, ubiquitylation; HID, homeobox-interacting domain; SRS, speckle-retention signal; AID, auto-inhibitory domain; MeCP, methyl-CpG-binding protein 2; CtBP, C-terminal binding protein; CBP, cAMP-response element-binding protein (CREB)-binding protein; ZBTB4, zinc-finger and BTB domain containing 4.

The family of homeodomain-interacting protein kinases (HIPKs) is evolutionally conserved and consists of four related kinases, HIPK1 to HIPK4 [29,30]. HIPK2 was first identified as an interactor with homeodomain transcription factor Nkx1.2 [29]. HIPK2 contains a 330 amino acid-long serine/threonine kinase domain located at the N-terminus of the molecule (Figure 2). The C-terminal part contains an interaction domain for the homeodomain transcription factors (HID), homeobox-interacting domain) and a binding motif for a small ubiquitin-like modifier (SUMO) in the speckle-retention signal (SRS) (Figure 2). The SRS is essential for the subcellular localization of HIPK2 in nuclear bodies. The C-terminal end of HIPK2 contains an interaction domain for the homeodomain transcription factors and an auto-inhibitory domain (AID) [31].

Studies of *Hipk1* and *Hipk2* single knockout mice suggest functional redundancy between HIPK1 and HIPK2 [32,33]. However, *Hipk1/Hipk2* double knockout mice are embryonically lethal and show defects in neural tube closure, hematopoietic cell differentiation, vasculogenesis, and angiogenesis [34]. HIPK2 has been classically identified as a nuclear kinase that acts as a corepressor for the homeodomain transcription factors [29]. Recently, HIPK2 was recognized as a signaling transducer transductor that is involved in a variety of intracellular signal pathways, including p53, transforming growth factor (TGF)-β, Notch, Wnt, JNK, Hedgehog, and Hippo [35,36,37,38,39,40]. In response to genotoxic stress, HIPK2 phosphorylates downstream substrates to regulate signaling of cellular development, cell cycle, cell proliferation, differentiation, and DDR [30,41,42]. HIPK2 binds and phosphorylates a large number of targets, including signal transducers, transcription factors, epigenetic regulators, and ubiquitin ligases (Table 1). Subsequently, it also associates with neurogenesis, myogenesis, angiogenesis, fat development, and hematopoiesis [37,43,44,45,46].

## 4. Regulation of HIPK2 Activities

HIPK2 functions are tightly coordinated by its catalytic activity, stability, and subcellular localization, which, in turn, are dynamically regulated by diverse post-translational modifications. In the absence of cellular stress, HIPK2 is generally maintained at low levels by ubiquitination-dependent proteasomal degradation mediated by E3 ubiquitin ligases, seven in absentia-homolog (Siah)-1 and tryptophan–aspartic acid (WD) repeats, and suppressor of cytokine signaling (SOCS) box-containing protein 1 (WSB-1) [51,55]. WSB-1 ubiquitinates the C-terminus of HIPK2 and thereby promotes proteasomal degradation [51]. Once cells are exposed to genotoxic stimuli, HIPK2 undergoes auto-phosphorylation and dissociates from WSB-1, thus protecting HIPK2 against proteasomal degradation [63]. Promyelocytic leukemia protein (PML) is a tumor suppressor that mainly localizes to punctate nuclear structures that are called PML nuclear bodies (PML-NBs). Fbx3 is a component of the Skp1-Cullin 1-F-box (SCF) E3 ligase complex in PML-NBs. HIPK2 is constitutively degraded by Fbx3-mediated polyubiquitination under normal conditions [64]. Increased stability and SUMOylation of PML protein in response to DNA damage mediates HIPK2 activation and induces cell death [65]. Siah-1 is another E3 ligase that mediates HIPK2 degradation in the absence of stress. In response to lethal DNA damage, ATM is activated and phosphorylates Siah-1 at Ser19, resulting in the stabilization of HIPK2 by escape from phosphorylated Siah-1 [55].

Hofmann et al. [65] have demonstrated that SUMO protease SuPr-1 interacts with HIPK2 and deconjugates SUMO from HIPK2, thereby antagonizing the inhibitory effect of SUMO-1 on HIPK2-mediated JNK activation. Thus, HIPK2 function on JNK and its p53-independent growth suppressing activity is modulated by reversible SUMO-1 conjugation and deconjugation. SUMOylation at K25 of HIPK2 is important for low level expression under normal conditions. Attachment of SUMO1 to K25 of HIPK2 is promoted by the SUMO E3 ligase Proprotein convertase 2 (Pc2) [53]. In unstressed cells, SUMOylated HIPK2 recruits histone deacetylase (HDAC) 3 and maintains HIPK2 in a deacetylated state. Exposure to oxidative stress mediates HIPK2 deSUMOylation and acetylation at K10 residues by cAMP-response element-binding protein (CREB)-binding protein (CBP) acetyl-transferase [66]. The changes of HIPK2 modification from SUMOylation to acetylation alters intracellular distribution of HIPK2 from nuclear speckles to the nucleoplasm. This subsequently relieves HIPK2-mediated transcriptional repression of several redox-sensitive genes, such as heme oxygenase 1 and peroxiredoxins [67,68]. Thus, proper acetylation of HIPK2 is essential for tolerance against sublethal DNA damage. Since HIPK2-mediated phosphorylation of Pc2 is required for its activation in response to genotoxic stress, Pc2, in turn, SUMOylates HIPK2, which then participates in the transcriptional repression of proapoptotic genes, such as *BAX*, to inhibit apoptosis and promote cell survival [53].

## 5. Roles of HIPK2 in DDR

HIPK2 is a DNA damage-responsive kinase that activates downstream targets, including tumor suppressor p53, which is often mutated or functionally inactivated in various types of cancer. The two master DNA damage checkpoint kinases, ATM and ATR, directly switch on HIPK2 upon different kinds of DNA damage by triggering its stabilization [55]. Exposure to IR is one of the genotoxic stresses which causes various types of complex clustered DNA damage, including DSBs, SSBs, oxidized bases, and nucleic mutations [5,69]. The multi-pathway of DNA repair mechanisms is triggered depending on the doses of IR to determine cell fate. p53 Ser46 phosphorylation plays a crucial role in IR-activated apoptotic programs. Dauth et al. [70] found that accumulation and activation of HIPK2 are significantly correlated with IR-induced phosphorylation of p53 Ser46. In addition, activation of ATM is required for IR-mediated HIPK2 accumulation. Thus, HIPK2 is the IR-triggered p53 Ser46 kinase and its function is regulated through DNA damage checkpoint kinase ATM. In addition, c-Abl, a tyrosine kinase which also activated by ATM, phosphorylates HIPK2 and this leads to HIPK2 accumulation and phosphorylation of p53 in response to γ- and UV-radiation, resulting in promoted apoptosis [71]. HIPK2 is an important modulator that senses the severity of DNA damage. For instance, in unstressed cells, HIPK2 is constantly degraded by the ubiquitin-proteasome system. In response to genotoxic stress, HIPK2 is stabilized by dissociation of E3 ubiquitin ligases. DNA damage also activates HIPK2 through caspase-6-mediated cleavage at Asp916 and Asp977. Caspase-mediated processing and the consequent removal of the C-terminal AID results in an increased activity in p53 Ser46 phosphorylation [49]. Once the damage is irreparable, these cells can ultimately trigger the cell death response to eliminate damaged, potentially threatening cells. Phosphorylation of p53 at Ser46 by HIPK2 in response to lethal DNA damage activates apoptotic target genes, such as *PUMA*, *BAX*, *NOXA*, and *BID* [72]. MDM2 is one of the potent p53 negative regulators. HIPK2 phosphorylates MDM2 and induces its proteasomal degradation, which leads to restoring p53 apoptotic activity [56]. In addition, HIPK2 can promote p53-independent apoptosis through interaction with C-terminal binding protein (CtBP) and Δp63α. CtBP is an anti-apoptotic transcriptional corepressor that inhibits cell death. UV-triggered CtBP phosphorylation at Ser422 by HIPK2 induces protein degradation in p53-null cells which promotes apoptosis [35]. The anti-apoptotic p63 isoform Δp63α is also phosphorylated by HIPK2 and targeted for degradation in a p53-independent manner [47]. In contrast, when DNA damage is less severe, repair is achieved by the DNA repair system in association with the required cell cycle arrest. In this case, HIPK2 does not affect phosphorylation of p53 at Ser46. Instead, HIPK2 mediates p53 recruitment onto the *CDKN1A* promoter through acetylation of p53 by p300/CBP-associated factor to induce cell cycle arrest followed by DNA repair [73]. HIPK2 resides in the nucleus where it partially co-localizes with the PML-NB [14]. PML-NB is essential for HIPK2-mediated p53 Ser46 phosphorylation and stabilization for the apoptosis-inducing function of HIPK2 after DNA damage [74]. In addition, the integrity of PML-NB is also regulated by HIPK2-dependent PML phosphorylation. During early stages of DNA damage, HIPK2 phosphorylates PML at Ser8 and Ser38 to increase stability of PML [75]. The deacetylase Sirtuin 1 (SIRT1) suppresses cell death after DNA damage by antagonizing acetylation of p53 [76]. Conrad et al. found that DNA damage initiates interaction between SIRT1 and HIPK2, which phosphorylates SIRT1 at Ser682 in response to lethal damage [54]. Phosphorylation of Ser682 inhibits SIRT1 activity in p53 acetylation and impacts expression of apoptotic p53 target genes and apoptosis. Thus, in response of severe DNA damage, HIPK2 strictly regulates SIRT1 activity through phosphorylation of SIRT1 in the PML-NBs. Thus, HIPK2 functions in the DDR by regulating cell cycle arrest and apoptosis, thereby helping to prevent mutations, genomic instability, and carcinogenesis.

## 6. HIPK2 as an Epigenetic Regulator

**Figure 3:**
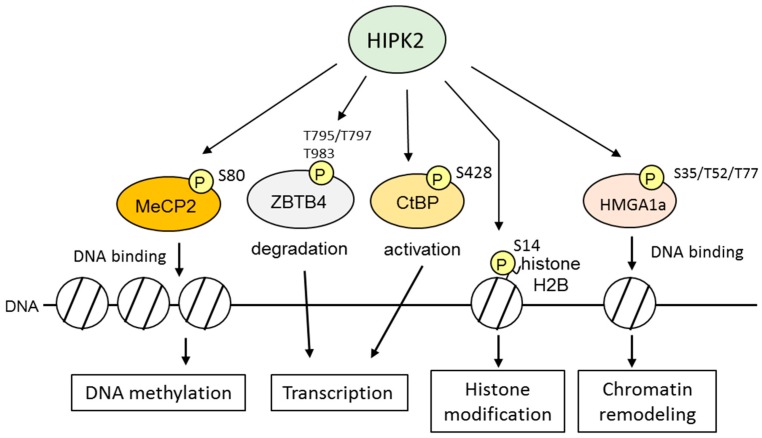
HIPK2 targets in epigenetic regulation. HIPK2 phosphorylates and activates epigenetic factors which are involved in DNA methylation, histone modification, chromatin remodeling, and transcription. Phosphorylated sites (P) by HIPK2 are shown.

Several studies have suggested that HIPK2 has a novel function as an epigenetic regulator of chromatin structure (Figure 3). For example, HIPK2 contributes to cell proliferation during cytokinesis through the phosphorylation of histone H2B at Ser14 [50]. A loss of H2B phosphorylation at the midbody caused by HIPK2 depletion prevents cell cleavage and tetra- and polyploidization. In addition, HIPK2 associates with chromatin modification factors, including methyl-CpG-binding protein 2 (MeCP2), methyl-binding transcription factor Zinc finger, and BTB domain-containing 4 (ZBTB4), transcriptional corepressor CtBP, and polycomb protein Pc2 [35,53,59,60]. Chromatin components and epigenetic factors promote DNA damage signaling and repair by regulating the integrated response of chromatin remodeling. MeCP2 represses transcription by its association with methylated DNA and recruitment of co-repressor proteins [77]. Phosphorylation of MeCP2 at Ser80 mediated by HIPK2 is required for DNA binding activity [59]. ZBTB4 also binds methylated DNA in vitro and in vivo and represses methylated sequences [78]. In response to DNA damage, HIPK2 phosphorylates threonine residues of ZBTB4 and accelerates its degradation [60].

**Figure 4:**
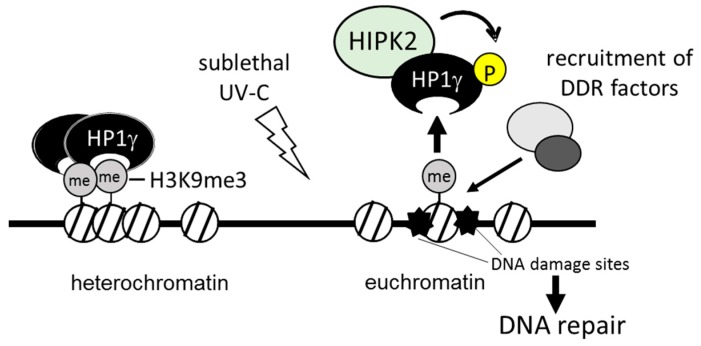
Schematic summary of HIPK2-mediated chromatin modification. Heterochromatin protein 1γ (HP1γ) binds to the methylated histone H3 and forms heterochromatin. In response to sublethal stress, HIPK2 phosphorylates HP1γ and facilitates its release from histone and promotes recruitment of DDR factors to the damaged sites for DNA repair. UV-C, ultraviolet-C.

Heterochromatin protein 1γ (HP1γ) consists of three orthologs, HP1α, HP1β, and HP1γ [79]. HP1 proteins are heterochromatin components that are associated with histone H3 di- or trimethylated at Lys9 (histone H3K9me2 and histone H3K9me3, respectively) through their N-terminal chromo domain [80]. HP1 proteins dimerize through their C-terminal chromo-shadow domain to promote heterochromatin formation, which induces gene silencing [79]. In addition, HP1 proteins associate with a number of chromatin modification proteins, including transcription regulators, DNA replication and repair-related proteins, via the chromo-shadow domain [80]. In response to genotoxic stress, chromatin structure is remodeled to increase the accessibility of the DDR machinery to the DNA damage sites. Several studies have demonstrated that HP1 proteins are released from histone H3K9me3 and facilitate recruitment of DDR factors to damaged sites, resulting in progression of DNA repair [81]. HP1 proteins are recruited again to DNA damage sites after they are released from chromatin [82]. These results suggest that the HP1 family may be involved in regulation of the chromatin structure by changing their binding affinity to chromatin in response to DNA damage and mediate recruitment of DNA repair factors to the damaged lesions. HIPK2 is specifically bound to HP1γ through its conserved pentapeptide motifs (883-PTVSV-887) called HP1box [62]. This association mediated phosphorylation of HP1γ in response to sublethal UV-C irradiation that resulted in facilitating the dissociation of HP1γ from histone H3K9me3 (Figure 4). UV-C induces pyrimidine–pyrimidone (6–4) photoproducts (6–4PPs), a major class of DNA lesions, which are removed by the nucleotide excision repair system [83]. The remaining photoproducts induces DSBs and then the surrounding histone H2A.X is phosphorylated at Ser139 (γ-H2AX) to initiate recruitment of DNA repair factors to DNA damaged sites. Knockdown of *HIPK2* impaired the removal of 6–4PPs and enhanced accumulation of γ-H2AX after UV-C irradiation [62]. This suggests HIPK2-dependent phosphorylation of HP1γ may regulate the dynamic interaction between HP1γ and chromatin for DNA damage repair. These data show a novel HIPK2-mediated pathway for DDR through the phosphorylation of chromatin remodeling factors.

## 7. A Role for HIPK2 in Notch1-Associated Tumorigenesis

After HIPK2 was first reported to induce p53 apoptotic activity through Ser46 phosphorylation, a number of studies uncovered additional molecular mechanisms for HIPK2-mediated p53 regulation and other downstream transcription factors that participate in cancer progression [84]. HIPK2 is now known to be a crucial regulator of DNA damage signaling and tumor suppression. A recent study identified Notch1 as a crucial downstream target of HIPK2. HIPK2 facilitates Fbw7-dependent proteasomal degradation of Notch1 by phosphorylating its intracellular domain (Notch1-IC) within the Cdc4 phosphodegron (CPD) motif. Under genotoxic stress, HIPK2 phosphorylates the residue T2512 in Notch1-IC and keeps it at a low level through proteasomal degradation [85]. Notch1 signaling is aberrantly activated in breast cancer. Moreover, increased expression of Notch1 intracellular domain (Notch1-IC) is associated with poor survival in patients with breast cancer and other tumors. Breast cancer tissues have increased Notch1-IC levels and show decreased levels of phosphorylated Notch1-IC T2512, HIPK2, and Fbw7. However, these changes are absent in cells expressing mutated Notch1-IC (T2512A), as well as the somatic mutants Notch1-IC P2513L and Notch1-IC P2515 frameshift. This is because these mutations block the HIPK2-induced phosphorylation of Notch1-IC and increase Notch1 stability. These studies have uncovered a novel mechanism for Notch1-dependent cancer progression and suggested an important role of HIPK2-induced phosphorylation of Notch1-IC at the T2512 residue in cancer prevention.

## 8. Pathophysiological Functions of HIPK2

Cellular responses to DNA damage are crucial to protect cells from genomic instability. Recent studies reveal that the DDR is correlated with immune response signaling networks and works together to maintain the harmonized multi-cellular function [86]. For instance, it is well established that viral genetic material triggers immunity of host cells by directly inducing cytokines, especially interferons (IFNs). Recent studies have documented that viral infection induces systemic DNA genetic and epigenetic alterations, including an increased frequency of homologous recombination (HR) [86]. There is increasing evidence that the DDR pathway is activated by microbial infection in humans. IFNα/β promotes p53 in turn evoking cell death that is crucial for antiviral immunity, thus, suggesting a novel relationship between IFNs and p53 signaling pathway [87]. Moreover, recent studies demonstrate that ATM modulates nuclear factor (NF)-κB activity through the activation of IκB kinases [88] and the phosphorylation of NF-κB at Ser547 [89]. Thus, ATM plays a key role as a hub of the DDR and immune response. In HIV-infected renal tubular epithelial cells, HIPK2 induced expression of pro-fibrosis markers via activating p53, TGF-β, and Wnt/Notch pathways [84]. Downregulation of HIPK2 leads to upregulated C-cadherin and decreases epithelial to mesenchymal transition [90]. These results suggest that HIPK2 is one of crucial regulators of kidney fibrosis by activating the upstream fibrosis signaling pathway. The tumor-suppressing functions of HIPK2 have been investigated to reside in its p53-dependent and p53-independent proapoptotic signaling transduction. In addition, HIPK2 is implicated in cytokinesis and prevents tetraploidization by phosphorylating histone H2B [50]. Failure in cytokinesis, the final step in cell division, causes chromosomal instability, a hallmark of cancer. Hence, HIPK2 contributes to cell proliferation and protects cells from tumorgenesis by controlling cytokinesis.

## 9. Conclusions

Although HIPK2 was originally thought to be a corepressor of a transcription factor, a large amount of data has shown that it also participates in diverse cellular activities, including a pro-apoptotic response to genetic damage by its serine/threonine kinase activity. Cellular responses to DNA damage are important to protect cells against genomic instability. The fundamental role of HIPK2 is acting as an integrating sensor for diverse cellular signaling. HIPK2 mediates different signaling pathways depending on dosages or types of the stimuli through its post-transcriptional modifications, subcellular localization, kinase activity, and substrate specificity results in the determination of cell fates. In recent years, increasing evidence reveals that the coordinated maintenance of the genome and epigenome in response to DNA damage is regulated by interactions between DDR factors and chromatin modifiers. Future studies could clarify the functions of HIPK2 in epigenetic regulation to promote DNA repair and genome stability. Finally, the contributions of HIPK2 to tumor regression and the response to anticancer drugs suggest that HIPK2 might be a diagnostic marker and a therapeutic target.

## Figures and Tables

**Table 1:** Phosphorylation targets of Homeodomain-Interacting Protein Kinase 2 (HIPK2) and functional roles.

Target Proteins	Cellular Function	Reference
p53	cell cycle regulation and apoptosis	[13,14,33]
ΔNp63α	cell cycle regulation and apoptosis	[47]
p27	cell cycle regulation and cell motility	[48]
PML	cell proliferation	[49]
H2B	cytokinesis	[50]
WIP1	DNA repair	[51]
PDX1	pancreatic development and mature β-cell function	[52]
β-catenin	activation of Wnt/β-catenin signaling	[36]
Pc2	SUMOylation	[53]
SIRT1	acetylation	[54]
Siah1, Siah2	ubiquitination	[55]
MDM2	ubiquitination	[56]
C/EBPβ	activation of transcription factor	[57]
CtBP	transcription corepression	[35]
p300, AML1	transcription activation; hematopoiesis	[44]
Che-1	transcription activation; cell proliferation	[58]
DAXX	transcription repression	[40]
MeCP2	DNA methylation and epigenetic regulation	[59]
ZBTB4	transcription and epigenetic regulation	[60]
HMGA1a	transcriptional regulation and chromatin remodeling	[61]
HP1γ	epigenetic regulation	[62]

PML, promyelocytic leukemia protein; WIP1, wild-type p53-inducible phosphatase 1; PDX1, pancreatic and duodenal homeobox 1; Pc2, Proprotein convertase 2; SIRT1, sirtuin 1; Siah-1, seven in absentia-homolog 1; C/EBPβ, CCAAT/enhancer binding protein β; AML1, acute myeloid leukemia 1; DAXX, death domain associated protein; HMGA1a, high mobility group AT-hook 1a; HP1γ, heterochromatin protein 1γ.
